# Expect the Worst! Expectations and Social Interactive Decision Making

**DOI:** 10.3390/brainsci11050572

**Published:** 2021-04-29

**Authors:** Cinzia Giorgetta, Alessandro Grecucci, Michele Graffeo, Nicolao Bonini, Roberta Ferrario, Alan G. Sanfey

**Affiliations:** 1 Department of Psychology and Cognitive Sciences, DipSCo, University of Trento, Corso Bettini, 84, 38068 Rovereto, Italy; alessandro.grecucci@unitn.it (A.G.); michele.graffeo@gmail.com (M.G.); 2 Centre for Medical Sciences, CISMed, University of Trento, Via S. Maria Maddalena, 1, 38122 Trento, Italy; 3 Department of Economics and Management, University of Trento, Via Vigilio Inama, 5, 38122 Trento, Italy; nicolao.bonini@unitn.it; 4 Institute of Cognitive Sciences and Technologies, CNR, Via alla Cascata 56 C, 38123 Povo, Italy; roberta.ferrario@cnr.it; 5 Donders Institute for Brain, Cognition and Behavior, Radboud University Nijmegen, 6525 AJ Nijmegen, The Netherlands; a.sanfey@donders.ru.nl; 6 Behavioural Science Institute, Radboud University Nijmegen, 6525 AJ Nijmegen, The Netherlands

**Keywords:** expectations, social decision-making, Ultimatum Game, Dictator Game

## Abstract

Psychological studies have demonstrated that expectations can have substantial effects on choice behavior, although the role of expectations on social decision making in particular has been relatively unexplored. To broaden our knowledge, we examined the role of expectations on decision making when interacting with new game partners and then also in a subsequent interaction with the same partners. To perform this, 38 participants played an Ultimatum Game (UG) in the role of responders and were primed to expect to play with two different groups of proposers, either those that were relatively fair (a tendency to propose an equal split—the high expectation condition) or unfair (with a history of offering unequal splits—the low expectation condition). After playing these 40 UG rounds, they then played 40 Dictator Games (DG) as allocator with the same set of partners. The results showed that expectations affect UG decisions, with a greater proportion of unfair offers rejected from the high as compared to the low expectation group, suggesting that players utilize specific expectations of social interaction as a behavioral reference point. Importantly, this was evident within subjects. Interestingly, we also demonstrated that these expectation effects carried over to the subsequent DG. Participants allocated more money to the recipients of the high expectation group as well to those who made equal offers and, in particular, when the latter were expected to behave unfairly, suggesting that people tend to forgive negative violations and appreciate and reward positive violations. Therefore, both the expectations of others’ behavior and their violations play an important role in subsequent allocation decisions. Together, these two studies extend our knowledge of the role of expectations in social decision making.

## 1. Introduction

Decision making is a vitally important cognitive ability that we are called upon to use on a daily basis. Decisions are often taken in what might be called an individual context, that is, when only the decision makers themselves are impacted by the outcome of the choice options, for example, a standard consumer choice. There is, however, a growing interest in a different class of decisions, namely, those taken in social interactive situations where the outcomes are often the product of the decisions of multiple involved agents (e.g., when people must negotiate about allocation of resources). These ‘social’ decisions [1,2] have received much less experimental investigation than individual decisions [3,4,5,6,7,8,9,10]. A particularly intriguing aspect to this latter set of decisions is the role of other factors that can affect choice in these contexts [11,12]. Several such factors have been studied, such as the impact of emotions, beliefs, and the preferences of others, with the role of expectations recently receiving much interest. Within this framework, the aim of this study is to investigate how the expectations about the behavior of others can affect choices in the context of social-interactive situations.

### 1.1. Role of Expectations in Decision Making

Psychological studies have demonstrated an important role for expectations on judgments and decisions. These studies have shown that different features, such as brands, pricing, and emotions, can impact expectations, which in turn can alter people’s behavior.

For example, expectations about vacations have been shown to affect people’s post-vacation evaluations [13], with similar effects reported for prior expectations about movies and the post-evaluation of their quality. The same applies for establishing norms [14]. Additionally, expectations of a beer’s brand identity [15] or constituent ingredients [16] can impact taste judgments. Further, Shiv at al. [17] demonstrated that expectations, via pricing information, can affect experiences of the efficacy of a product. Here, participants who paid full price for an energy drink reported receiving a higher benefit from this product as compared to those who paid a discounted price for the same drink. Relatedly, Jing and Duo [18] demonstrated that the expectations based on sausage brand (well known vs. less known) affect the prediction and memory of its taste and choice.

Interestingly, the effects of expectations have been also examined in social decision-making contexts such as negotiation decisions. For example, in a negotiation task Van Kleef et al. [19] showed that participants’ choices were influenced by the emotions of their opponents. Indeed, emotions and their expressions influence not only the self but also the behavior of others during social interactions. For example, it has also been demonstrated that a negotiator is more willing to have a business relationship with those that display positive emotions, whereas displaying negative emotions lead negotiators to be more demanding [20].

In the field of neuroeconomics, Delgado et al. [21] has shown that in classic economic games, such as the Trust Game, decisions are influenced by prior knowledge about one’s partner. Here, before interacting with others, participants were provided with information about the personality of these partners. Results indicated that this did indeed have a bearing on how the participants made decisions about the partner, namely, if the player was a ‘good’ person, then bad game behavior was more readily forgiven. Thus, these findings show that prior social knowledge about a particular partner can influence learning from actual observed behavior, showing a clear ‘top-down’ influence on social decision making. Other experiments have also highlighted that people are more willing to invest trust in partners perceived, via facial expression, as more trustworthy than untrustworthy [22]. Several studies [23,24,25,26] demonstrated, in the context of social interaction, how our behavior is guided by expectations concerning others’ behavior and choices. In particular, one study [23] showed that people’s choices are affected by empirical expectations (that is, choices one believes others would actually make). Additionally, [24] demonstrated that the likelihood of pro-social behavior is increased by observing others behaving pro-socially. More recently, Bicchieri and colleagues [26] claimed as the exposure to the social environment and social behaviors around us influence our social norms and then they affect our decision making. The role of norms has been largely studied in decision making, and their influence has been recently clarified [14,27].

Finally, a recent study [28] investigated whether the activation of an expectation system in one domain would influence (by facilitating or reducing) the consequences of expectations in another domain, in other words, whether there exists a ‘domain-general expectancy mechanism’. More specifically, here the authors studied the effects of an unexpected melody on the participants’ sensitivity to the violations of fairness and behavioral reactions to that (by the willingness to punish those who behaved unfairly). Even though the results showed that participants punished less when the melodies were unexpected, the findings from this study failed to clearly understand the expectation process.

Taken together, these results highlight that expectations can indeed modulate decision making. However, in most of these studies the participants have constructed expectations themselves, with direct manipulations of expectation rather rarely used.

### 1.2. The Role of Explicit Manipulation of Expectations in Social Decision-Making Contexts

One study [29] has explicitly manipulated the general expectations of players in a social interactive task and then directly observed the consequences on their behavior. In this study, an Ultimatum Game paradigm [30] was used. In this task, two players are given the opportunity to divide a certain amount of money provided by the experimenter, usually EUR 10 or USD 10. One player is named the Proposer, while the other the Responder. The Proposer decides how much money to keep for herself and how much will go to the Responder. Thus, the Proposer makes an offer, which can broadly be categorized as either fair (e.g., EUR 5 to Responder, keeping EUR 5 for herself) or unfair (e.g., EUR 1 to Responder, keeping EUR 9 for herself). The Responder then has to decide whether to either accept or reject the offer. If the responder accepts the offer, then the money is split as proposed; if the responder rejects the offer then, neither player receives anything. According to standard economic theory game, the Proposer should offer the lowest possible amount of money, and in turn the Responder should accept this offer because any money is preferable to no money. However, a vast amount of studies (see [31] for a summary of findings) have shown that this is atypical behavior. Indeed, the Proposer’s offers are usually around 50% of the total amount of money; the Responder rejects low offers (corresponding to < 20% of the total amount available) about 50% of the time.

Although several theories have been proposed suggesting that the offers are evaluated from an equity perspective (i.e., dividing 50/50) with rejections occurring when participants are ‘inequity-averse’ [32,33,34], other studies have demonstrated that factors other than the division itself can play important roles, for example, preexisting or incidental emotional states [4,5,6,7,8,9,35,36,37,38] can lead to changes in acceptance rates. Of interest here is the Sanfey [29] study, which showed that expectations play a crucial role in rejection rates in the UG. In this study, expectations were manipulated directly in a between-subjects design by informing different groups of Responders that UG offers were typically either equal or unequal (although in reality both groups saw an identical set of offers), in addition to a group who saw no expectation information. The results showed that expectations did indeed influence Responder behavior, with players primed with higher expectations rejecting significantly more offers than those with low or no prior expectations. This study clearly highlights that people use specific expectations regarding social interaction as a behavioral reference point and that information about the players we are partnered with can have a significant impact on social decision making.

One limitation of this study, however, was the question of whether expectations can impact decision behavior in both directions (lower and higher) in a within-subjects design, that is, with the same player receiving information about different types of Proposers. This is important as we live in a highly interactive environment, where we generally interact with different classes of people. By employing a between-subjects design, as in the aforementioned study, participants are aware only of one potential pattern of behavior (i.e., one-third of participants knew that proposers generally make equal offers, one-third knew that proposers would make unequal offers, and one-third received no information), which may bias choice. Instead, by using a within-subjects design, we can examine whether players may flexibly shift decision behavior in accordance with particular expectations. More specifically, the within-subjects design exposes participants to different proposer strategies, those of equal share or unequal share, respectively, and this information will help address the question of whether different decision rules are used by the same individual based on differing expectations. In previous studies, there was only one expectation set, and the participants therefore had no reason to invoke different choice rules. Moreover, a within-subjects design allows for assessing individual differences (e.g., inequity aversion, incidental emotions) which can impact the decisions themselves. Therefore, in order to address these issues, we employed an Ultimatum Game paradigm to examine whether different expectations of others’ behavior impact decision making, hypothesizing that our findings will confirm and extend those of Sanfey [29].

### 1.3. The Role of Expectations on Subsequent Decisions in Social Contexts

The vast majority of UG experiments focus on so-called one-shot versions of the task, where each Proposer is encountered only once. However, as we live in interactive environments, it can often happen that we have to make subsequent decisions in both, individual, e.g., [36,37,39,40] and social contexts, e.g., [41,42,43,44,45,46,47,48,49,50]. Of specific interest here, in social contexts, we may encounter the same person again, and we may then have to decide how to treat this person in this subsequent interaction. Therefore, it is interesting to examine how the combination of expectations and prior behavior taken together predict future interactions with the same individual. In order to answer this second question, we used the Ultimatum Game behavior as described above as the basis for studying how prior expectations outcomes impact subsequent decision making when encountering the same game partner again, this time in a Dictator Game.

Previous studies have highlighted the human ability to detect, remember, and punish ‘cheaters’ [41]. People appear better at remembering actions associated with cheating rather than trustworthy behaviors [42,43,44], and related studies have shown that conditional reasoning improves when participants are asked to detect violations rather than non-violations of a social contract [45,46]. However, other studies on cheater detection have shown somewhat different results. For example, a study [48] demonstrated that memory improves for altruistic behaviors, and other studies have failed to find any differential memory abilities related to the behavior of others [47,48,49]. Therefore, the results regarding memories of remember players as a function of their prior behavior are mixed. One study [50] potentially accounted for these variable findings by showing that the key factor in determining whether a partner would be remembered or not is not their behavior per se, but rather whether this behavior was in accordance with the participants’ expectations or not. Here, UG Proposers were remembered better when their offers violated the participants’ expectations, irrespective of the offer itself.

This existing literature on the role of expectations on social interaction has therefore primarily investigated how expectations affect first-time interactions for partners already met, but none of these studies investigated whether and how expectations interact with prior behavior again encounter the same person that we have previously interacted with. Therefore, an interesting question worth addressing is the following: Is social decision making affected by our expectations when we meet a person for the second time and must decide how to treat them? More specifically, are these decisions affected more by our prior expectations or by the way these people actually behaved in a previous meeting or by the interaction between the two? Additionally, do negative and positive violations differently affect subsequent behaviors? Answering these questions will enable us to understand the role of social dynamics and expectations in interactive social decision-making situations.

In order to address this second question, we added a Dictator Game paradigm to follow the Ultimatum Game. As with the Ultimatum game, in the Dictator game [51] there are two players that have to divide a sum of money (usually EUR 10 or the equivalent). One player is the allocator who can decide how much money to keep and how much to offer to the second player, termed the recipient. In contrast to the Ultimatum game, the recipient must accept whatever the allocator decides to share. Thus, the allocator freely makes a proposal without the uncertainty of whether the offer will be rejected, and as such the DG is thought to be a better measure of ‘pure’ altruism [52]. Therefore, the second aim of the present study is to investigate how people treat partners with whom they have already interacted and about whom they have been primed with expectations. Players will play a DG in the role of allocator with the group of 40 partners they have previously encountered as Proposers in the UG. As this is the first study exploring these aforementioned questions, our hypotheses here are mostly exploratory. However, it is reasonable to assume that participants will have better memory of those Proposers who violated their expectations, as found in Chang et al. [50], and therefore in turn may allocate less money to those they expected to behave fairly but that instead made unfair offers. However, if participants preferentially encode altruistic behavior, as claimed by Barclay and Lalumiere [47], there may be enhanced memory for those who behaved fairly, regardless of expectations, and with greater subsequent allocations to these players.

By investigating how people behave when they act as responder in an Ultimatum Game and subsequently as allocator in a Dictator Game with the same partner, this study can usefully extend previous knowledge as to the role of expectations on social decision-making interactions.

## 2. Materials and Methods

### 2.1. Participants

Thirty-eight university students participated in this study (20 females; mean age: 25.2 ± 5.6 years). None of the participants had prior knowledge of the Ultimatum Game or Dictator Games. All participants had normal or corrected to normal vision and provided written informed consent. To ensure participants’ motivation, they were informed that they would be monetarily compensated depending on their choices along the experiment. In fact, for ethical reasons, each participant received the same amount of money (EUR 10) at the end of the study. The entire session lasted about 1 h.

### 2.2. Experimental Procedures

**Figure 1:**
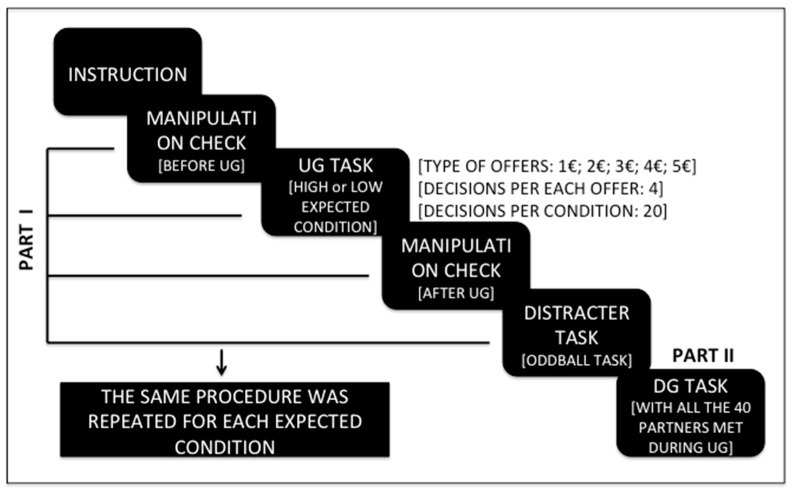
Experimental design.

We employed a within-subjects experimental design and utilized an Ultimatum Game and a Dictator Game, modified from previous standard Ultimatum Game [30] and Dictator Game [51] paradigms. After providing informed consent, the participants were introduced to the experiment. The participants played the Ultimatum Game first, followed by the Dictator Game. Before and after each UG round, the participants were asked to answer questions regarding their expectations. See Figure 1 for detail. See the Supplementary Material for the instructions used during the experiment.

#### 2.2.1. Part I: Ultimatum Game

##### Expectation Induction

To induce the relevant expectations, the participants were first provided with an instruction packet describing the Ultimatum Game task. They were asked to read a one-sentence description of Ultimatum Game rules. After that, they were informed how the game is usually played as follows: ‘Just to give you some information about how the game is typically played by normal players, in general there are some players whose most common offers are quite equal, that is, they offer 4 or 5 euro when dividing 10 euro; whereas there are other players, whose most common offers are quite unequal, that is, they offer 1 or 2 euro when dividing 10 euro. These two types of players have been separated into two different groups. You will first play with one group and then with the other. Before each round you will be informed which of these two groups of players you will be playing with’. A graphical representation of offers made by each group was also shown to the participants.

##### Expectation Induction Check (before Playing the Ultimatum Game)

After the expectation induction phase, but prior to game play, the participants were asked to estimate what range of offers they would expect proposers to make in the Ultimatum Game. This was carried out to assess the degree to which the expectation induction primed the participants.

The participants were asked to imagine they were playing with 100 players belonging to either the group of players that typically make equal offers (high expectation condition) or unequal offers (low expectation condition) and that the amount of the pot to be split was in each case EUR 10. They were asked to indicate how many offers, of the potential set from EUR 1 to EUR 5, they expected to receive when playing with each group.

##### Decision Task (Ultimatum Game)

Prior to playing the Ultimatum Game, the participants were provided with detailed instructions about the task. It was emphasized that all offers were pre-made and independent, that is, the participants’ decisions could not affect the offer of the following proposer. The participants always played as responder and received a proposal from a EUR 10 endowment to the proposer. The two experimental conditions, low and high expectations, were presented separately from each other, as two different blocks of offers. The sets of offers presented in each of the two blocks were identical. Within each block, participants received 20 offers each offer coming from a different human proposer and each of which they had to decide to either accept or reject. The offer set consisted of four repetitions of each of five possible offers (EUR 5, EUR 4, EUR 3, EUR 2 and EUR 1 out of EUR 10). The offer type and partner pictures associated with each offer were fully randomized within each block and for each participant. The pictures were selected from a pool of Ultimatum Game players’ photographs extensively used in previous studies [38,53]. We chose 40 different pictures, with an equal proportion of males and females; all faces were Caucasian, and all had emotionally neutral expressions.

The order of presentation of the two expectation conditions were thus blocked and were also counterbalanced across participants. Therefore, half of the participants played first with low expectation players and then with high expectation players, and the other half of participants saw the opposite order. This was performed in order to prevent any order effects between the two different conditions. The game was played in its computerized version using the e-Prime software package.

**Figure 2:**
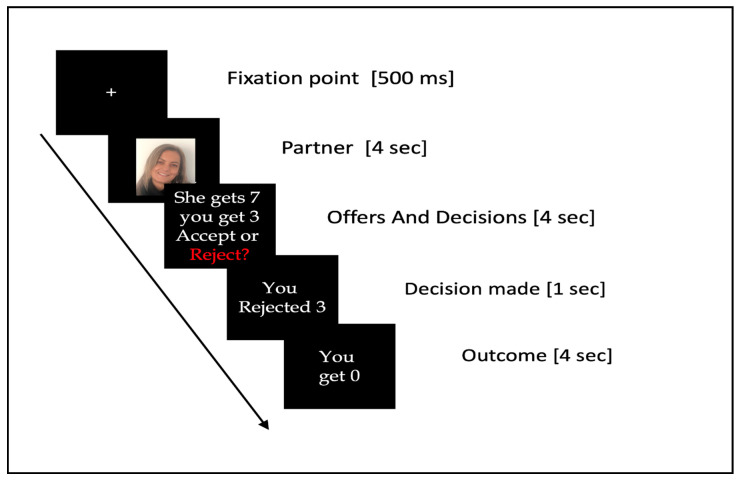
Ultimatum Game task.

In each trial, participants first saw a fixation cross for 500 ms, followed by a picture of the proposer in that trial for 4000 ms, and then the proposer’s offer for a further 4000 ms. During the offer display, participants had to decide to either accept or reject the offer by pressing the corresponding button on a computer keyboard. The decision was then displayed for 1000 ms, and finally the outcome was presented for 4000 ms (see Figure 2).

##### Expectation Modification Check (after Playing the Ultimatum Game)

At the end of each block of the Ultimatum Game, participants were asked again to estimate the range of offers they would expect from that group of proposers if they would play a UG round with them. This was carried out separately for both the high expectation and low expectation groups after the corresponding run. This enabled an assessment of the degree to which the expectations were modulated by the actual proposer behavior experienced by the participants.

##### Distracter Task

After each block, the participants were asked to perform an oddball detection task, which here acted as distracter task. The participants were presented with two different strings of numbers (11111) and (99999). In one condition, the two strings of numbers were presented, respectively, 80% (11111) and 20% (99999) of the times. In the other condition, the percentage of presentations was reversed. In each condition, there were 100 trials in total. The two conditions were presented randomly. Participants were instructed to count how many times they saw the string of numbers which appeared more often and to indicate its total at the end of the task.

#### 2.2.2. Part II: Decision Task (Dictator Game)

After the distracter task, the participants played several Dictator Games. First, the rules of the Dictator Game were fully explained, and then participants were told they would play in the role of allocator and that the various recipients would be the same set of players they had previously been paired with in the Ultimatum Game. Each round involved a division of EUR 10, and participants could offer any amount from EUR 0 to EUR 10 to their game partner, retaining the remainder for themselves. It was made clear that the other player could not accept or reject the offer but would simply receive the allocated amount. All proposers from the previous UG were presented in a single round, with participants therefore playing a total of forty trials.

**Figure 3:**
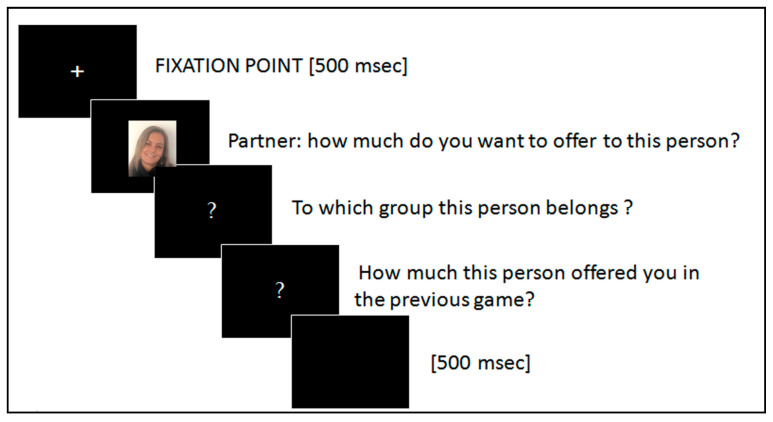
Dictator Game task.

In each trial, the participants first saw a fixation cross for 500 ms, followed by the picture of the recipient for that trial. They then indicated how much they wanted to allocate to that recipient. After that, they were asked to indicate to which of the previous groups the recipient had belonged when he/she played the role of proposer (fair or unfair offer), and how much they thought that he/she had previously offered them in the Ultimatum Game. Responses were provided by using the computer keyboard. The three questions were self-paced, and the participants were told they would be paid a percentage of what they kept for themselves during the dictator game (see Figure 3).

## 3. Results

Statistical analyses were performed using STATISTICA and the R package “ImerTest”.

### 3.1. Expectation Induction Check (before Playing)

To begin with, we examined the effectiveness of the primary experimental manipulation. We compared participants’ expectations of Ultimatum Game offers following the induction of low and high offers, respectively. We performed this by calculating, as per Sanfey [29], the average offer that participants reported expecting to receive if playing with 100 independent proposers in each of the two experimental conditions. The offers amount expected were calculated, per each participant, as follows: (([A × EUR 5] + [B × EUR 4] + [C × EUR 3] + [D × EUR 2] + [E × EUR 1])/100), where A, B, C, D, and E are the numbers of proposers expected to offer, respectively, EUR 5, EUR 4, EUR 3, EUR 2, and EUR 1. Therefore, if a participant expected to receive, for example, EUR 5 from 50 players, EUR 4 from 20, EUR 3 from 10, EUR 2 from 10, and EUR 1 from 10, his/her calculated expected offer amount was of EUR 3.90.

A one way ANOVA analysis, with expectations (high vs. low) as the independent variable and the expected offer amount as the dependent variable, confirmed that experimental manipulation was successful (F(1, 37) = 373.46, *p* = 0.001). Participants expected to receive substantially higher offers when playing with the high expectation group (M = EUR 0.11 ± EUR 0.55), than when playing with the low expectation group (M = EUR 1.87 ± EUR 0.40).

### 3.2. Ultimatum Game Decisions

**Figure 4:**
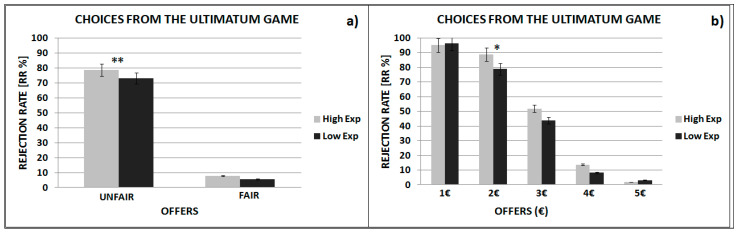
(**a**) Average of rejection rates for unfair and fair offer sets respectively. (**b**) Average of rejection rates for all offers. Line bars indicate, in both graphs, standard errors of the average of rejection rates. * *p* value = 0.05, ** *p* value = 0.02.

In order to compare our results to those reported in previous studies [29] we collapsed the two most equal offers (EUR 4 and EUR 5) and the three most unequal offers (EUR1, EUR 2, and EUR 3) into two categories (namely, fair and unfair). The 2 (expectations: high vs. low) × 2 (offers: fair vs. unfair) within-subjects ANOVA demonstrated both main effects of the offer (F(1, 37) = 372.72, *p* = 0.001) and expectations (F(1, 37) = 5.44, *p* = 0.02), as well as a significant interaction effect (F(1, 37) = 6.26, *p* = 0.02) (see Figure 4a). Post hoc analyses, Bonferroni corrected, showed that participants rejected more unfair offers when playing with the high expectation group than when playing with the low expectation group (*p* = 0.001 for all the comparisons). No differences between the two expectation conditions were found for fair offers (*p* = 1).

To specifically examine the effect of expectations on individual offers, we conducted a linear mixed model (LMM) with a step-wise procedure. Before performing these analyses, we checked whether rejection rates were affected by the order of the presentation of expected conditions for all our experimental conditions: high vs. low expectation group by fair vs. unfair offers. The results did not show any significant effect (*p* > 0.05 for all the comparisons), and therefore we decided not to take any more into consideration for this factor. We proceeded as follows: First, we included expectations (high vs. low), then the offer amount (EUR 5, EUR 4, EUR 3, EUR 2, EUR 1), and finally their interaction. The subject was modeled as a random factor in order to check for individual differences, with the rejection rate as the dependent variable. The findings showed a significant effect for expectations (*z* value = 1.92, β = 0.20; *p* = 0.05), as well as a significant effect of offer amount (Chi-square(4) = 1209.8, *p*-value = 0.001), and a significant interaction (Chi-square(4) = 9.3, *p*-value = 0.05). These findings highlighted that the participants rejected more offers in the high than low expectation condition, with this particularly true for the EUR 2 offers (see Figure 4b). The individual differences were also significant (Chi-square(1) = 159, *p*-value = 0.001).

We performed additional analyses (see the Supplementary Material for more details), where we were able to exclude any effect of each single decision on the subsequent decision, in both the expectation conditions of the Ultimatum Game. We also explored, and reported in the Supplementary Material, the distribution of choices among subjects.

### 3.3. Expectation Induction Check (after Playing)

We also compared the participants’ expectations of UG offers after having played the game and, thus, after exposure to the offer sets. To compute the overall expectations for each participant we followed the same procedure as outlined above. ANOVA analyses again showed a significant effect of expectation manipulation (F(1, 37) = 28.99, *p* = 0.001). The participants still expected to receive higher offers from the high expectation group (M = EUR 3.41 ± EUR 0.59) than from the low expectation group (M = EUR 2.66 ± EUR 0.55), even after seeing the identical set of offers from each group.

We further compared whether there were differences in expectations before and after playing the Ultimatum Game. A 2 (predictions: before vs. after playing) × 2 (expectations: high vs. low) ANOVA analysis was performed, revealing a main effect of expectations (F(1, 37) = 218.49, *p* = 0.001) and an interaction effect between predictions and expectations (F(1, 37) = 88.66, *p* = 0.001). Post hoc analysis showed that participants had higher expectations before playing as compared to after playing in the high expectation condition, whereas they had lower expectations before playing than after playing in the low expectation condition (*p* = 0.001 for both comparisons). Therefore, although there was still an effect of expectation prime on the future estimates of the offer amounts, this was moderated by the effect of actually experiencing a more neutral set of offers (with a EUR 3 mean).

### 3.4. Dictator Game Decisions

#### 3.4.1. Memory and Allocation Behavior in the DG

With the aim of investigating the respective roles of expectations and experience on subsequent social decision making, we analyzed the ability to remember the previous encountered player, during the Ultimatum Game, and the money allocated to him/her during the Dictator Game and their interaction. We also checked for individual differences in each measure and their interaction.

First, we performed a linear mixed model (LMM) with memory for the previous proposer as the dependent variable and expectations (high vs. low) and offer amount (from EUR 1 to EUR 5) as within-subjects independent variables. We found a main effect of expectations (*z* value = 3.53, β = 3.82; *p* = 0.001), demonstrating that participants had better memory for the proposers belonging to the high expectation condition (M = 57.5% ± 12.5%) than to the low one (M = 53.7% ± 13%). Individual differences were also significant (Chi-square(1) = 80.6, *p*-value = 0.001).

Next, in order to analyze the allocation amount to the recipient as a function of their behavior as proposer in the previous UG, we collapsed UG offers of EUR 1, EUR 2, EUR 3, EUR 4, and EUR 5 across both the high and low expectation conditions. Therefore, there were four game partners for each UG offer amount (i.e., EUR1–5) per each expectation condition. Then, we performed a linear mixed model (LMM) by using expectations (high vs. low) and offer amount (from EUR 1 to EUR 5) as within-subjects factors. The results showed a main effect of expectations (z value = 2.71, β = 1.25; *p* = 0.007), such that participants allocated more money when playing with recipients belonging to the high UG expectation group (M = EUR 16.1 ± EUR 3.38) than to the low UG expectation group (M = EUR 15.6 ± EUR 3.67). Neither the main effect of the offer amount nor the interaction between expectations and offers were significant. Individual differences were instead significant (Chi-square(1) = 339, *p*-value = 0.001).

Finally, we also checked for the interaction between the memory performance and the behavior shown in the UG. For this, we performed LMM with memory (remembered vs. not remembered), expectation (high vs. low), and offer amount (from EUR 1 to EUR 5) as factors, with allocation as the dependent variable. The findings showed that the interaction between memory and expectation, and also the three-way interaction, were not significant (*p* >.05). However, the model showed that there were still significant individual differences (Chi-square(1) = 333, *p*-value = 0.001).

#### 3.4.2. Role of Memory on the Allocation Behavior at the DG

**Figure 5:**
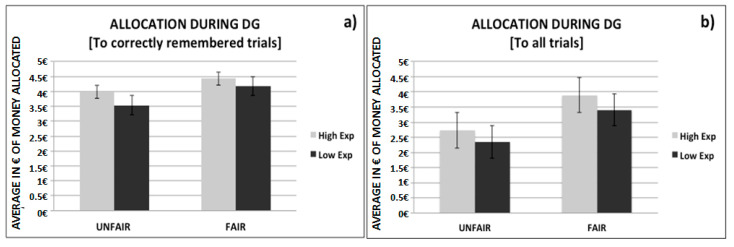
(**a**) Average amount of money the participants allocated only to the correctly remembered trials. (**b**) Average amount of money allocated in all DG trials, as a function of remembered offers and expectation. Line bars indicate the standard errors of the mean (of money allocated).

In order to examine how participants behaved with the recipients they had previously encountered (in the role of proposers) and remembered, we performed further analyses wherein we calculated the average allocation only when the participants correctly remembered the group to which the recipient belonged by taking into consideration the offer he/she made as proposer during the UG. Specifically, we split this group into the two more equal (EUR 4–5) and the three more unequal (EUR 1–3) offers. Then, we performed a 2 (offers: fair vs. unfair) × 2 (expectations: high vs. low) ANOVA analysis on only correctly remembered trials, with the dependent variable the average amount of money allocated in the DG. Our analyses yielded significant main effects for both offer (F(1, 21) = 9.89, *p* = 0.005) and expectation (F(1, 21) = 4.2, *p* = 0.05). Specifically, the participants allocated more money to the recipients who previously made fair offers as compared to unfair offers and to players belonging to the high rather than to the low expectation condition. No interaction effect was found (F(1, 21) = 0.13, *p* = 0.72) (See Figure 5a).

To further examine the role of prior experience on the participants’ behavior in the DG, we analyzed the allocation they made based on what they remembered (both group type and UG offer amount for each recipient) regardless whether it was correct or not (Figure 5b). We performed a 2 (subjective reported offers: fair vs. unfair) × 2 (subjective reported expectations: high vs. low) ANOVA analysis on all the trials. The dependent variable was the average amount of money allocated.

Here we found significant main effects for both subjective reported offers (F(1, 20) = 31.97, *p* = 0.001) and subjective reported expectations (F(1, 20) = 7.64, *p* = 0.05), meaning that participants allocated more money to the recipients that they believed made fair offers than those they thought made unfair offers, but also to recipients who they believed belonged to the high as opposed to the low expectation condition. There were no significant interaction effects [F(1, 20) = 0.19, *p* = 0.67].

#### 3.4.3. Mismatch between Expectations during UG and Allocation Behavior at the DG

In order to check whether memory and allocation behavior were affected by the expectations about the Ultimatum Game offers, we compared data between the trials where the offers matched (M) the expectations (that is, offers of EUR 4–5 in the high expectation condition and offers of EUR 1–3 in the low expectation condition) and where they did not match (nM) the expectations (offering EUR 4–5 in the low expectation condition and EUR 1–3 in the high expectation condition).

With this aim, we performed two LMM analyses by using as dependent variables, respectively, the memory and allocation amount and, as factors, the expectation match (matched vs. not matched) and actual expectations themselves (high vs. low).

**Figure 6:**
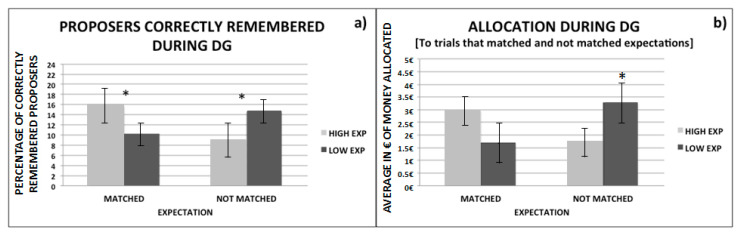
The percentage of correctly remembered proposers and the average amount of money allocated during the DG to recipients when they (proposers/recipients) matched and did not match prior high and low expectations are reported, respectively, on the left and right panels. Line bars indicate respectively: (**a**) standard errors of average of correctly remembered proposers, (**b**) standard errors of average of the money allocated. * *p* value = 0.001.

With regard to the memory results (see Figure 6a), our findings showed a significant effect for the main factors of expectation match (*z* value = 6.42, β = 2.18; *p* = 0.03) and expectation (*z* value = 9.53, β = 3.09; *p* = 0.003) and a significant interaction effect between the two (*z* value = 15.7, β = 3.93; *p* = 0.001). Specifically, the participants better remembered the proposers that made the more equal offers (EUR 4 and EUR 5), regardless of whether they did or did not match their expectations.

With regard to the allocation during the DG (see Figure 6b), the results showed significant effects for both expectation match (*z* value = −4.50, β = −1.20; *p* = 0.001) and expectation (*z* value = −5.04, β = −1.26; *p* = 0.001) and a significant interaction between them (*z* value = 7.14, β = 2.78; *p* = 0.001). Taken together, these findings show that participants allocated more money to recipients who previously, as proposers, made unexpectedly fair offers, as compared to both those who made unexpectedly low offers, as well as to those who made low offers as expected. This finding suggests that people are more willing to reward those who previously behaved better than expected.

## 4. Discussion

In this study, we investigated how explicit expectations of social behavior impact economic decision making in interactive contexts. Specifically, we aimed to address two main questions: Firstly, we focused on understanding the role of the expectations regarding proposer behavior when playing a standard Ultimatum Game in the role of responder. Secondly, we expanded the investigation beyond this immediate interaction to also examine the role of expectations on subsequent decisions when encountering a game partner (in the role of recipient in the DG) who had previously behaved either fairly or unfairly (in the role of proposer in the UG).

To date, there have been relatively few studies [19,20,21,22,25,29] that have investigated the role of expectations in social decision-making contexts. Importantly, with only a few exceptions these studies have generally not used explicit manipulation of expectations, which makes an assessment of how expectations can influence decision behavior in social interactions more difficult given that the evidence to date has been largely correlational. Further, the studies that did use explicit manipulations, e.g., [29] have used a between-subjects design, whereby different groups of participants received different sets of expectations about their respective proposers. We were interested in whether these findings would extend to a within-subjects design, that is, where responders might decide differently about the same monetary offer coming from different types of proposers. Notably, knowledge about what to expect from a given proposer is not particularly useful information in the UG—the offer is actually explicitly made to the responder, with the latter’s decision being simply to either accept or reject that specific offer. Therefore, the expectation should be largely irrelevant to this accept/reject decision, at least according to standard economic theories of decision making, such as Utility Theory.

Our findings showed that the experimental manipulation was effective in this within-subjects condition. Our findings clearly showed that these expectations affected decision behavior. The participants rejected unfair offers at a higher rate when these were made by a group that was expected to make equal, as opposed to unequal, offers. That is, the proposers for whom the participants had high expectations were punished more severely when making low offers, presumably because participants perceived these offers as more unfair.

In line with previous evidence showing that responders in the UG like to be treated fairly and punish those who behave unfairly [4,5,6,7,9,10,53,54,55], our study highlights that unequal offers are perceived as even more unfair when they violate the expectations we hold about the proposer making them, which leads in turn to greater rejections. Interestingly, we found that this was true, as in Sanfey [29], for all the three most unequal offers (that is, EUR1, 2, and 3 out of EUR 10), but also that this occurred mostly when faced with offers of EUR 2. Therefore, our findings highlight not only that expectations play a pivotal role in social decision making, independent of the experimental context (between- or within-subjects condition), but that these expectations are most effective when there is greater ambiguity about the nature of the offer itself. As has been shown in previous studies, e.g., [4,7], offers of EUR 2 are perceived as more ambiguous in terms of their inherent fairness, and thus our data suggest that expectations are primarily relevant in driving our choices in more ambiguous situations. That is, expectations primarily help the responder to interpret an offer as fair or not when its fairness status is not clear, and in turn, help make the related decision. Expectations make little difference in the rejection rates when the offer is unambiguous (e.g., EUR 1, EUR 3, EUR 4, or EUR 5). Overall, despite finding individual differences in UG behavior across participants, congruent with our hypothesis, expectations still played a pivotal role in driving the choices.

These findings are also in line with other studies [56,57,58], which show that expectations affect the decision-making process, both from a behavioral and neural perspective. More specifically, in the allocation decision-making task, deviation from our expectations is directly related to both a higher reaction time and to greater brain activity in the anterior insula (AIns) and anterior cingulate cortex (ACC), regardless of whether it is an advantageous or disadvantageous deviation for the participants from the expected equal outcome. Additionally, these studies confirm the fact that establishing a norm can effectively substitute for weak enforceable restrictions [59]. Indeed, norms can be a powerful way to constrain decisions [14]. Other results have shown that people do not have stable preferences but change equity evaluation in different situations [33].

With regard to the role of expectations on subsequent decision making, while research has investigated our ability to remember social partners who treated us either well or poorly, e.g., [41,42,43,46,47,60] and, more recently, how economic interactions and expectation violations affect subsequent memories of interaction partners [50], none of these studies, to the best of our knowledge, have investigated how these interactions and expectations affect the way people subsequently behave in future contexts. With this aim in mind, we employed (unannounced in advance) a standard Dictator Game following the Ultimatum Game, in which participants played the role of allocator and where the recipient had previously played as proposer in the Ultimatum Game.

Our findings showed that, in general, proposers from the high expectation group were better remembered than those from the low expectation group. When comparing allocations in the Dictator Game with the actual offers made by the two groups of previous proposers, the results demonstrated that the participants allocated more money to recipients of the high expectation group than to those of the low expectation group, regardless of the offers they actually made, and also to those who made fair offers, regardless of the group they belonged to. Of course, these data are based on implicit memory. The allocations made to recipients who had been correctly remembered exhibited similar results. A possible explanation for these findings can be found in the consumer choice research domain, where it has been found that people’s choices are to some degree also affected by when the choice is made, e.g., [61]. For example, Milkman, Rogers and Bazerman [62] showed that should-food (e.g., vegetables) was chosen more than want-food (e.g., ice cream) when the purchase is delayed with respect to the choice. There was a similar relationship between the two economic games used in this study. In the Ultimatum Game, which occurred soon after expectation was induced, participants decided to reject unfair offers more often, whereas in the Dictator Game, which occurred later with respect to expectation induction, participants act more as they ‘should’ act, that is, offering more to the proposers who made fair offers and to those belonging to the high expectation group (regardless of the offers they really made). Future studies can usefully further investigate this interaction between expectation induction and delay on time of the choices. One limitation of our study is that the DG always followed the UG, so we cannot assess if similar or different effects could have happened when inverting the sequence. Future studies can explore the issue of the spill-over effects across different tasks [11,12]. Moreover, although the number of subjects used in this study is in line with the literature, e.g., [4,6,7,9,53], in future studies it could be useful to collect a higher number of subjects for each single condition as a possibility for enhancing power effects.

We also aimed to examine the role of violated expectations when participants actually remembered proposers. Thus, we analyzed memory for UG proposers and subsequent DG allocation. We performed this by comparing the conditions where proposers matched and did not match expectations. The results demonstrated that proposers were remembered more often when they had made fair offers, regardless of whether this behavior was expected. Regarding the allocation behavior, participants gave more money to recipients who in the previous UG made, as proposers, unexpectedly fair offers than to those who made unfair offers, regardless of whether the latter were expected. Taken together, these results support our second explorative hypothesis; that is, people prefer to reciprocate generous behavior. Moreover, our findings are in line with [27], who demonstrated the importance of prior ownership in bargaining over a jointly produced surplus. The results suggest that proposers respect prior ownership (a form of expectation).

Following Chang and Sanfey [50], we might have found enhanced memory for proposers who violated initial expectations. However, our experiment differs from this previous study in several important ways. There, participants were presented with 24 proposers’ faces they had previously encountered as well as 24 new faces, and they were asked to indicate their confidence about whether they had interacted with him/her before. In the present study, only previously encountered proposers’ faces were presented. Since these studies are the only two investigating this question by using such economic games, future studies can further investigate these aspects.

It is worth pointing out here that our findings clearly highlight how people are driven by reciprocal behavior. Memory improves not only for proposers who are expected to make equal offers regardless of the real offers they made, but also for those who actually made equal offers, regardless of whether this behavior was expected. Moreover, when a player belongs to a fair expected group, he/she is forgiven even when he/she acts unfairly. When a player belongs to an unfair expectation group, but he/she acts fairly, his/her behavior is appreciated and more money is allocated to him/her, as compared to those who behaved unfairly as expected. In other words, negative violations lead to being forgiven, and positive violations are instead appreciated and then reciprocated. These findings support and extend those found by Barclay and Lalumiere [47], and overall, clearly suggest that not only expectations but also their violations play a pivotal role in also driving subsequent decisions during social interaction.

Previous studies have shown that in the Dictator Game some allocators decide to keep all the money, some give less than half, and others prefer to split it equally, e.g., [61,63,64,65,66,67], with these behaviors driven by a variety of motivations. In this study we have been able to show the important role played by expectations in allocators’ altruistic behavior.

In addition to these suggestions, such as the concern for reciprocity [68], personal sense of shame [69], and the desire to spite [70], in this study we have shown that both the expectations of others’ behavior and also their violations play a crucial role in human interactive decision making.

## 5. Conclusions

In conclusion, we believe these results are important as they clarify two main points: the possibility of affecting human interactive decision making based on the expectations of the partner in a first interaction and the effect of both expectations and their violations in a subsequent interaction.

The results show in a within-subjects study that expectations affect decision-making behavior by increasing punishment behavior, e.g., rejecting more of the low offers of participants who were expected to act in a more altruistic way.

In the subsequent interaction, the proposers who were expected to behave fairly were better remembered than those expected to behave unfairly. Moreover, more money was allocated to recipients belonging to the high expectation group regardless of their real behavior. These findings thus suggest that people tend to forgive negative violations and reward positive violations.

Our study extends previous findings on this topic and highlights, for the first time, that both the expectations of others’ behavior and also their violations play an important role in subsequent decisions. Therefore, this study sheds new light on how, in social interactions, people utilize specific expectations as a reference point.

## Data Availability

The datasets generated during and analyzed during the current study are available from the corresponding author on request.

## Supplementary Materials

The following are available online at https://www.mdpi.com/article/10.3390/brainsci11050572/s1, Table S1: Difference in accepted offers and number of participants. Table S2: Differences of accepted offers for each amount of money. Table S3: Number of participants that chose differently across conditions.
