# Supplementary material for: Expect the Worst! Expectations and Social Interactive Decision Making
Source: Brain Sci. 2021 Apr 29;11(5):572. doi: 10.3390/brainsci11050572 (PMC8145296; doi:10.3390/brainsci11050572)
Supplement: Supplementary file 1 [file brainsci-11-00572-s001.zip › brainsci-1099349-supplementary.pdf]

SUPPLEMENTARY FILE

1) INSTRUCTIONS USED DURING THE EXPERIMENT

The experiment was conducted at the Laboratory of Decision Science at the Department of Psychology and Cognitive Science of the University of Trento. It was conducted during the day, between 10 a.m. and 6 p.m. All participants were students at the University of Trento. More specifically, the majority were students at the Department of Psychology and Cognitive Science and had previous experience as participants in various experiments. However, none knew either the Ultimatum Game or the Dictator Game.

When participants entered the lab they were first instructed on the Ultimatum Game and after playing it, they were then instructed on the Dictator Game. They were initially informed that the experiment had three different phases (UG, oddball, and DG) and that they would be instructed before each phase about what they had to do.

Specifically:

1. **Instructions for the Ultimatum Game**

   As reported in the manuscript, subjects were given the instructions for playing the UG and about the expectations:

   "In each trial you will play with a different partner. You will only see his/her picture and the assigned number but not his/her name, in order to keep the partner anonymous. In each trial, the partner (proposer) will offer you some of the 10 euro, according to his or her decision. What you have to do, as responder, is to decide whether to accept or reject the offer. If you accept the offer then money will be split as proposed, if you decide to reject it then neither you nor the proposer will get any money. Be careful because if you don't answer then the proposer will get the money he/she decided to keep but you will not receive anything. If you accept the offer then you have to press the "n", if you reject it then you have to press the "m" on the keyboard of the computer. It is really important that you take into consideration that the proposer can neither see the offer made by the other proposers and nor know your answer to the other offers. Therefore, the offers of each proposer are not affected by what happened earlier. At the end of the game, you will be paid according to your decisions during the game. That is, you will receive a fixed amount of money for your participation at the study, and you

will receive a percentage of the amount you have really earned during the game. Now we will start with a few practise trials, which will better explain how to play".

2. **Instructions for the Dictator Game**

   After the UG part, participants were instructed to the DG as follow:

   "Now you will play with the same players you met during the UG game but this time you will be the one who can decide how much to keep and to offer to the other player. Moreover, the other player cannot decide whether to accept or reject the offer, but will just receive the money you decided for offer to him/her. After each choice you will be asked to indicate to which group that player belonged. If he/she belonged to the group that made equal offers then you will have to press the "n", if instead he/she belonged to the group that made unequal offera then you will have to press the "m". After that, you will be asked how much that player offered you in the UG. You can answer by using the numbers on the keyboard. As in the UG, at the end of the DG you will be paid a percentage of the total amount you decided to keep for yourself. If the instructions are clear then press the spacebar on the keyboard. Now we will start with a few practise trials, which will better explain how to play".

2) ADDITIONAL ANALYSES

Here we report additional regressions in order to investigate further the effect of each single decision on the subsequent decisions. These analyses enabled us to exclude any effect of trial by trial decisions, and of their distribution across conditions. For example, in order to investigate the change in acceptance rate across various offer repetitions we considered the influence that a decision has on the choice to accept or reject the offer with the same monetary value. We have run a regression model to test if choice "n+1" (i.e. the choice to accept or reject the second, third or fourth offer for each offer level) was influenced by the previous choice "n" (i.e. the first, second or third choice for each offer level). Therefore, this analysis uses factors of the choice (accept vs. reject), the level of the offers (1-5€) and the experimental condition (H vs. L), plus the three second-order interactions. The regression analysis on this model indicates a significant main effect of the level of offers, that is, a significant difference between the €1 offers and the €3, €4 and €5 offers ($\beta = -1.78823$; $p < 0.001$, $\beta = -2.23715$; $p < 0.001$ and $\beta = -2.52398$; $p < 0.001$, respectively) and a main effect of the choice ($\beta =$

1.33705; p = 0.005). This suggests that the number of rejected offers is significantly lower for higher offers compared to the €1 offer, and also that participants tend to repeat the same choice, that is, if they reject an offer (e.g. the first one) they usually also reject the following iterations of the same offer level. We can conclude that each decision has a carry-over effect, that is, it influences the subsequent choices. No significant main effects for the experimental condition and for the interaction effects have been found.

In conclusion, these additional analyses highlighted that participants were quite consistent in their choices, and that repeated exposure to the same offer and its distribution did not affect their decisions, and that this was true independent of the expectation conditions. It is also important to remember here that participants knew that the behaviour of the Proposers was not influenced by the choices they had previously made, and additionally that offers were presented in a fully randomized order. Therefore, there was, in our opinion, relatively little room for distribution effects in this experiment.

Additionally, in order to further investigate the distribution of choices among our subjects, we quantified how players reacted to the experimental manipulations. First of all, we calculated the total number of differences, i.e. the number of offers of the same monetary value that were accepted in one condition and not in the other. Then we calculated how many participants made different choices between the conditions (see the following table):

Table S1. Difference in accepted offers and number of participants.

Difference in accepted offers between the two conditions.	Number of participants (% of 38 participants)
0	12 (32%)
1	6 (16%)
2	7 (18%)
3	4 (11%)
4	3 (8%)
5	4 (11%)
6	1 (3%)
10	1 (3%)
Total differences between the conditions: 80	38 (100%)

The difference in accepted offers between high and low expectation vary across the 5 levels of the UG offer amounts. In the following table we report the number of differences for each offer amount. Note that, in agreement with results we showed in the paper, the majority of the differences is concentrated in the €2 and €3 offers:

Table S2. Differences of accepted offers for each amount of money

Type of offers	Difference in accepted offers between the two conditions.
€ 1	8 (10%)
€ 2	24 (30%)
€ 3	28 (35%)
€ 4	13 (16%)
€ 5	7 (9%)
	80 (100%)

Finally, we examined the number of participants (not the number of choices) that chose differently across the conditions, broken down by the offer amount.

Table S3. Number of participants that chose differently across conditions.

Type of offers	Number of participants that chose differently between the two conditions.
€ 1	6
€ 2	16
€ 3	16
€ 4	9
€ 5	6

Note: the number of participants is not equal to 38 because the same participant could accept a different number of offers in more than one level of offer.

Therefore, we can conclude that there are notable individual differences, in that not all subjects react in the same way to the same offers across the two experimental conditions. However, the means differences were consistent among the different experimental conditions taken into consideration.
